# Renal Cell Carcinoma Metastasis to the Gallbladder Detected by FDG-PET/CT

**DOI:** 10.14740/jocmr1886w

**Published:** 2014-09-09

**Authors:** Aung Zaw Win

**Affiliations:** Department of Radiology, San Francisco VA Medical Center, 4150 Clement Street, San Francisco, CA 94121, USA. Email: aungzwin@gmail.com

**Keywords:** Renal cell carcinoma, FDG-PET/CT, Gallbladder, Metastasis

## Abstract

A 62-year-old male presented to the ER with three episodes of diffuse abdominal pain which occurred after eating. He had a history of renal cell carcinoma (RCC), prostate cancer and bladder cancer. FDG-PET/CT scan showed a hypermetabolic soft tissue density within the fundus of the gallbladder. The patient underwent laparoscopic cholecystectomy and surgical pathology revealed clear cell type RCC. This is the first report that features PET/CT imaging to detect RCC metastasis to the gallbladder. Lesions within the gall bladder and their clinical manifestations can be non-specific and PET/CT can help characterize them. RCC metastasis to the gallbladder is very rare but it should be included in the differential diagnosis, especially in patients with a history of RCC.

## Introduction

Renal cell carcinoma (RCC) most commonly originates in the proximal convoluted tubules. Smoking and obesity are other known risk factors [1]. The incidence of RCC is eight times higher in highly developed countries, in comparison to developing countries [2]. It is two times more common in males than in females. The incidence and mortality of RCC is higher in African Americans than in whites [3]. Hematuria is the most common symptom of this type of cancer. Other symptoms include flank pain, abdominal mass, fever, cough and bone pain. Microscopically, clear cell tumors form solid sheets, trabeculae or glandular and papillary structures with abundant clear cytoplasm and central or eccentric hyperchromatic nuclei [4]. RCC has been proven to be a chemotherapy-resistant tumor [5]. RCC has a tendency of late recurrence and about 20-40% of patients develop metastases after radical nephrectomy [6]. Therefore, it is very important to monitor these patients with serial imaging studies for tumor recurrence in RCC. FDG-PET/CT can examine all organ systems in one procedure non-invasively without the need for contrast agents. Although FDG-PET has proved to be an invaluable tool in staging a variety of cancer types such as lung, breast, lymphoma, colorectal, and head and neck, it currently has a limited role in evaluating RCC.

## Case Report

**Figure 1:**
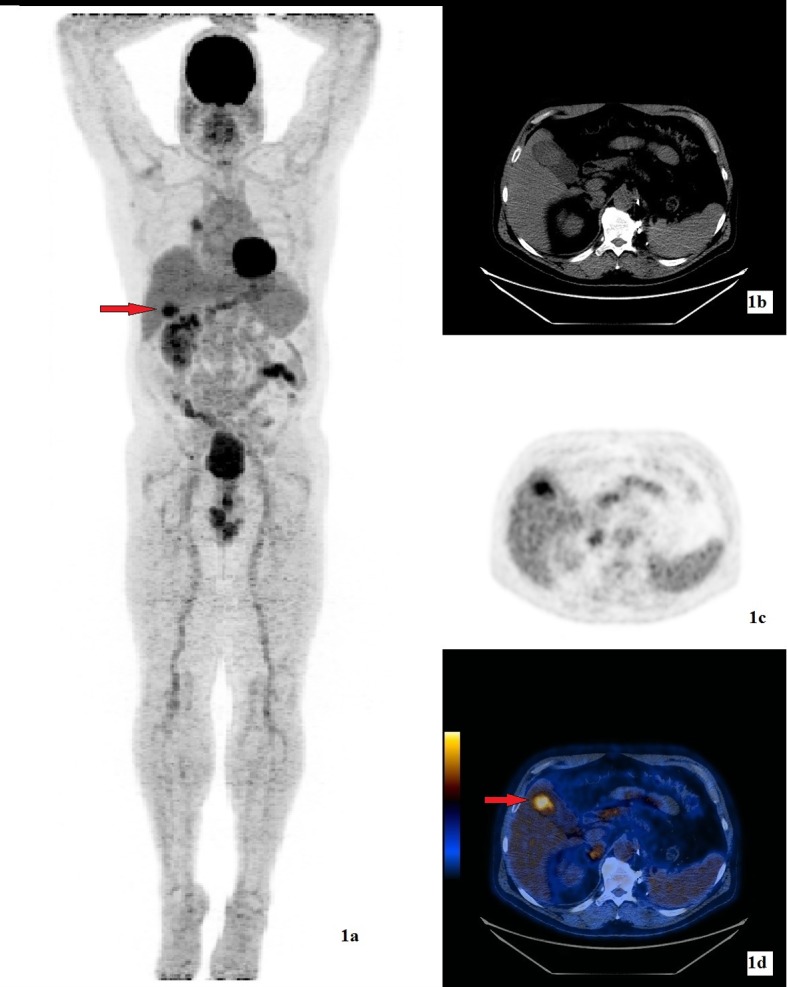
(a) 3D MIP image of FDG-PET/CT study showing a hypermetabolic lesion (arrow) in the gallbladder. (b) CT showing a non-specific hyperdense nodule within the gallbladder. The gallbladder was otherwise normal with no wall thickening and no pericholecystic fluid. (c) FDG emission image. (d) Hybrid FDG-PET/CT image showing a hypermetabolic soft tissue density of SUVmax 6 (arrow) within the fundus of the gallbladder.

**Figure 2:**
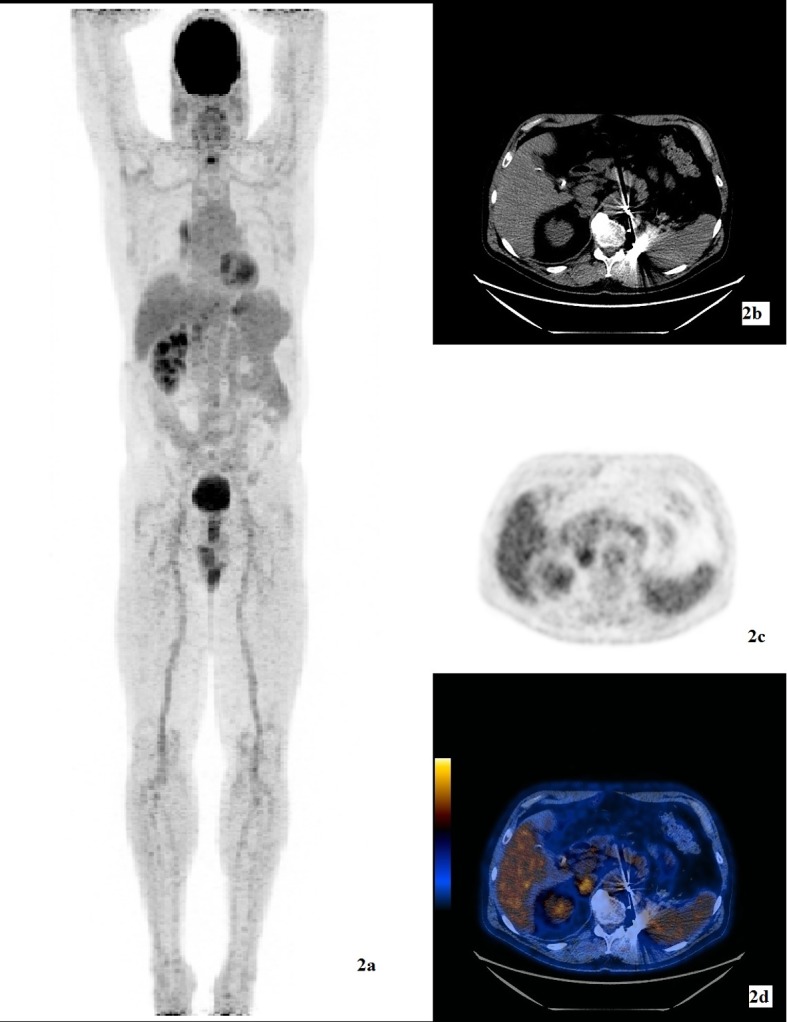
(a) 3D MIP image of FDG-PET/CT study after the laparoscopic cholecystectomy. (b) CT transmission image for attenuation correction and anatomic localization. (c) FDG emission image. (d) Hybrid FDG-PET/CT image showing inflammatory changes after laparoscopic cholecystectomy but no signs of local recurrence.

Our patient was diagnosed with clear cell type RCC at the age of 40, in 1999. He had no family history of cancer. He underwent left nephrectomy and left adrenalectomy and he was treated with 17 cycles of sutinimib. The RCC metastasized to the contralateral adrenal, upper lobe of right lung and left fifth rib. Consequently, he had cyberknife procedure to the right adrenal, and upper lobe of right lung. In April 2013, 21 years after the initial diagnosis of RCC, he presented to the ER with three episodes of diffuse abdominal pain which occurred after eating. He denied weight loss or change in appetite. There was positive Murphy’s sign on physical exam. Liver function test showed elevated AST 40 U/L (normal 5 - 35) and ALT 173 U/L (normal 7 - 56). He had a history of gall stones. FDG-PET/CT exam showed a hypermetabolic soft tissue density measuring approximately 4 cm in diameter with SUVmax 6 within the fundus of the gallbladder (Fig. 1). He underwent elective laparoscopic cholecystectomy. The differential diagnosis before the surgery was primary gallbladder carcinoma versus a secondary metastasis. Surgical pathology revealed clear cell type RCC, Fuhrman grade 3. The metastasis was limited to the mucosa of the gallbladder, with no invasion into or through the gallbladder wall. The first PET/CT exam after the cholecystectomy showed no signs of local recurrence (Fig. 2). Interval follow-up PET/CT exams are planned for this patient to monitor and restage his cancers.

## Discussion

This is the first report that features the PET/CT imaging to detect RCC metastasis to the gallbladder. This is also the first study to report the metastasis of RCC to bilateral adrenal glands and rib, in addition to gallbladder. Furthermore, this is the first case in which gallbladder metastasis is reported in a patient with a history of three types of cancer: prostate, bladder and RCC. Bilateral occurrence of RCC is found only in 0.5% of all RCC cases [5]. Our patient had RCC in both kidneys. Patients with distant metastases from RCC have a poor prognosis, with a 5-year survival rate of less than 5% [7]. Yet, our patient has been living with RCC for almost 22 years, despite the distant metastases. The common sites of RCC metastasis are lung, bone, liver and brain. Gallbladder involvement with RCC has been reported at a rate of less than 0.6% and this is usually detected only at autopsy [8, 9]. Clinical diagnosis of gallbladder metastasis is even rarer.

Our patient has a history of gallstones and primary gallbladder cancer is highly correlated with gallstone disease. Acute cholecystitis, commonly caused by gallstones, can also occur in gallbladder carcinoma. In addition, patients with gallbladder cancer can have similar presentations to those with chronic cholecystitis. Metastases to the gallbladder initially occur as small flat nodules in the mucosal layer and resemble a primary gallbladder carcinoma [10]. Clear cell RCC and clear cell carcinoid tumor of the gallbladder can occur at the same time in patients with Von Hippel-Lindau (VHL) disease and patients without VHL [11]. So, it is difficult to distinguish primary versus secondary gallbladder cancer on CT. Sand et al reported that RCC metastasis to the gallbladder follows the hematogenous route and the tumors are rarely greater than several millimeters in size [12]. Thus, it is a challenge for CT imaging to detect such small lesions.

A common CT appearance of gallbladder carcinoma is either focal or diffuse gallbladder wall thickening. However, diffuse wall thickening is very non-specific and it is also caused by a wide variety of benign pathologic conditions, such as acute and chronic cholecystitis, adenomyomatosis and low protein states. Another common CT appearance of gallbladder tumors is an intraluminal polyp. Gallbladder polyps are relatively common and found in up to 5% of the adult population [13]. Most polyps are benign. Cholesterol polyps and primary and secondary gallbladder carcinoma can all have this polypoid appearance. In this case, there was no polyp and instead, there was only a mass in the fundus of the gallbladder. Thus, gallbladder tumors have non-specific features on CT. Reiser et al wrote that it is difficult for CT and MRI to differentiate between benign and malignant gallbladder tumors [14]. Secondary gallbladder cancer is very rare, with the most frequent primary sources being malignant melanoma, colon, breast and pancreas [15]. The fundus of the gallbladder is a common site for RCC metastasis [15]. Cholecystectomy provides the best survival outcome for both primary and secondary gallbladder tumors [15].

Peak incidence of RCC is in the sixth decade of life but our patient was first diagnosed with RCC when he was 40. The most common presenting complaint of carcinoma of gallbladder is right upper quadrant pain. Our patient had acute presentation of diffuse abdominal pain and such physical symptoms are non-specific. Elevated alkaline phosphatase is often found with gallbladder cancer. However, in our case, the level of alkaline phosphatase was normal and AST and ALT levels were elevated. Our patient had Fuhrman grade 3 RCC metastasis and from the literature, Fuhrman grade has little association with gallbladder metastasis [8]. In the present case, the metastasis was confined to the mucosa of the gallbladder but there are cases which involve both the mucosa and the muscular layer [8]. RCC originating in either the right or the left kidney has equal probability of metastasizing to the gallbladder.

Chung et al found that gallbladder metastasis is associated with male gender, over 60 age group and clear cell type RCC [15]. Our case fits the findings from that study. Furthermore, according to the same study, most patients were asymptomatic, had pancreatic metastasis and the median time to gallbladder metastasis following nephrectomy was 4 years [15]. Our patient was unique in that the metastasis to the gallbladder occurred 21 years after the nephrectomy. The longer interval between diagnosis of RCC and gallbladder metastasis is associated with better survival [16].

It is difficult to obtain a preoperative diagnosis of gallbladder metastasis. It is possible for RCC to metastasize to any organ in the body, such as ovary, thyroid and skin [17]. Metastases can also occur via the lymphatic route [17]. FDG-PET/CT is a good tool because it can detect cancers even before anatomic changes are apparent. RCC metastasis to the gallbladder is very rare but it should be included in the differential diagnosis, especially in patients with a history of RCC. Currently, CT is the method of choice for detection and staging of RCC. FDG-PET/CT should be incorporated as a standard exam in the management of RCC.
