# Correction: Aung Zaw Win, Renal Cell Carcinoma Metastasis to the Gallbladder Detected by FDG-PET/CT

**DOI:** 10.14740/jocmr2146e

**Published:** 2015-03-01

**Authors:** Aung Zaw Win

**Affiliations:** Department of Radiology, San Francisco VA Medical Center, 4150 Clement Street, San Francisco, CA 94121, USA. Email: aungzwin@gmail.com

Corrections to article “Renal Cell Carcinoma Metastasis to the Gallbladder Detected by FDG-PET/CT”, by Aung Zaw Win, published in Vol. 6, No. 6, 2014, p482-486, doi: http://dx.doi.org/10.14740/jocmr1886w. The author would like to add a contributing author in the second position of author list, the new author list should read as follows.

Aung Zaw Win^a, c^, Carina Mari Aparici^b^

^a^Department of Radiology, San Francisco VA Medical Center, 4150 Clement Street, San Francisco, CA 94121, USA

^b^Department of Radiology, University California San Francisco (UCSF), 500 Parnassus Ave, San Francisco, CA 94143, USA

^c^Corresponding Author: Aung Zaw Win, Department of Radiology, San Francisco VA Medical Center, 4150 Clement Street, San Francisco, CA 94121, USA. Email: aungzwin@gmail.com

This work was performed at the San Francisco VA Medical Center, 4150 Clement Street, San Francisco, CA 94121, USA

